# LY6E: a conductor of malignant tumor growth through modulation of the PTEN/PI3K/Akt/HIF-1 axis

**DOI:** 10.18632/oncotarget.11670

**Published:** 2016-08-29

**Authors:** Chan Joo Yeom, Lihua Zeng, Yoko Goto, Akiyo Morinibu, Yuxi Zhu, Kazumi Shinomiya, Minoru Kobayashi, Satoshi Itasaka, Michio Yoshimura, Cheol-Goo Hur, Hideaki Kakeya, Ester M. Hammond, Masahiro Hiraoka, Hiroshi Harada

**Affiliations:** ^1^ Department of Radiation Oncology and Image-Applied Therapy, Kyoto University Graduate School of Medicine, Sakyo-ku, Kyoto 606-8507, Japan; ^2^ Group of Radiation and Tumor Biology, Career-Path Promotion Unit for Young Life Scientists, Kyoto University, Yoshida Konoe-cho, Sakyo-ku, Kyoto 606-8501, Japan; ^3^ Department of Radiation Medicine, Fourth Military Medical University, Xi'an, Shaanxi 710032, China; ^4^ Department of Oncology, The First Affiliated Hospital of Chongqing Medical University, Yuanjiagang, Yuzhong District, Chongqing 400016, China; ^5^ Cancer Genomics Branch, Division of Convergence Technology, National Cancer Center, Ilsandong-gu, Goyang-si, Gyeonggi-do 410-769, Korea; ^6^ Division of Bioinformatics and Chemical Genomics, Graduate School of Pharmaceutical Sciences, Kyoto University, Sakyo-ku, Kyoto 606-8501, Japan; ^7^ CRUK/MRC Oxford Institute for Radiation Oncology, Department of Oncology, University of Oxford, Oxford OX3 7DQ, United Kingdom; ^8^ Hakubi Center, Kyoto University, Yoshida-Honmachi, Sakyo-ku, Kyoto 606-8501, Japan; ^9^ Precursory Research for Embryonic Science and Technology (PRESTO), Japan Science and Technology Agency (JST), Kawaguchi, Saitama 332-0012, Japan; ^10^ Laboratory of Cancer Cell Biology, Radiation Biology Center, Kyoto University, Yoshida Konoe-cho, Sakyo-ku, Kyoto 606-8501, Japan

**Keywords:** tumor hypoxia, hypoxia-inducible factor 1 (HIF-1), lymphocyte antigen 6 complex, locus E (LY6E), PTEN/PI3K/Akt/HIF-1 axis

## Abstract

Lymphocyte antigen 6 complex, locus E (LY6E) has been implicated in the malignant progression of various types of cancers; however, the underlying mechanism remains unclear. Here, we identified LY6E as an activator of HIF-1 and revealed their mechanistic and functional links in malignant tumor growth. The aberrant overexpression of LY6E increased HIF-1α gene expression principally at the transcription level. This, in turn, led to the expression of the pro-angiogenic factors, *VEGFA* and *PDGFB*, through decreases in the expression levels of PTEN mRNA and subsequent activation of the PI3K/Akt pathway. The LY6E-HIF-1 axis functioned to increase tumor blood vessel density and promoted tumor growth in immunodeficient mice. LY6E expression levels were significantly higher in human breast cancers than in normal breast tissues, and were strongly associated with the poor prognoses of various cancer patients. Our results characterized LY6E as a novel conductor of tumor growth through its modulation of the PTEN/PI3K/Akt/HIF-1 axis and demonstrated the validity of targeting this pathway for cancer therapy.

## INTRODUCTION

Lymphocyte antigen 6 complex, locus E (LY6E), also designated as stem cell antigen 2 (SCA2) and thymic shared antigen-1 (TSA-1), is a member of the lymphostromal cell membrane Ly6 superfamily [1–3]. All Ly6 family members are low-molecular weight (10-12 kDa) proteins that display limited nucleotide and amino acid sequence homologies with one another, but commonly have a conserved cysteine-rich cell surface domain and are anchored to the cell surface *via* a glycosil-phosphatidylinositol (GPI) moiety [4]. Since the cloning of the mouse Ly-6E.1 and human LY6E cDNAs in 1986 and 1993, respectively, extensive efforts have been devoted to demonstrating their physiological importance in cell-cell adhesion [5], signaling transduction *via* the T cell receptor, and T cell development [6–9]. In the field of cancer biology associations with tumorigenicity [10–12], cancer stem cell properties [13], and cancer metastases [12, 14] have been described. However, it currently remains unclear how LY6E promotes malignant phenotypes and accelerates tumor growth.

Many solid tumors contain hypoxic regions due to an imbalance between the uncontrolled proliferation of cancer cells and insufficient formation of a vascular network. Cancer cells express many genes in a hypoxia-inducible factor-1 (HIF-1)-dependent manner in order to survive in hypoxic environments [15, 16]. HIF-1 is a heterodimeric transcription factor composed of an α-subunit (HIF-1α) and β-subunit (HIF-1β), and its activity is mainly dependent on the stability of the HIF-1α protein [16, 17]. Under normoxic conditions, the proline residues, P402 and P564, in the oxygen-dependent degradation (ODD) domain of HIF-1α are hydroxylated by prolyl-4-hydroxylases (PHDs) [18, 19]. This hydroxylation triggers the ubiquitination of HIF-1α by a von Hippel-Lindau (VHL)-containing E3 ubiquitin ligase, leading to the rapid degradation of HIF-1α [18, 19]. In contrast, HIF-1α becomes stable under hypoxic conditions because of the inactivation of oxygen-dependent hydroxylases, and then interacts with HIF-1β [18, 19]. The resultant heterodimer, HIF-1, binds to enhancer regions called the hypoxia response element (HRE) and induces the expression of a series of genes, such as vascular endothelial cell growth factor (VEGF) and platelet-derived growth factor B (PDGFB), at the transcriptional level [15, 16]. In addition to the oxygen-dependent regulation of HIF-1α stability, HIF-1 activity was previously shown to be regulated at multiple steps, such as the transcriptional initiation [20], translational initiation [21], and transactivation [22] of HIF-1α. Clinical as well as basic research has shown that HIF-1 is associated with angiogenesis, metabolic reprogramming, and the invasion and metastasis of cancer cells as well as the poor prognoses of cancer patients [15, 16], which justifies the targeting of HIF-1 itself or its upstream activators for cancer therapy [15, 23]. However, whereas the oxygen-dependent regulation of HIF-1 activity has been characterized, the upstream signaling pathways that stimulate HIF-1 activity have not yet been fully elucidated, which makes it difficult to develop strategies that efficiently inhibit HIF-1 activity.

In the present study, we provided a novel insight into the regulatory mechanism underlying tumor growth *via* activation of the LY6E-HIF-1 pathway. We successfully identified LY6E as a novel upstream activator of HIF-1 through a genetic screening strategy we recently established [24, 25]. The aberrant expression of LY6E was shown to promote tumor growth by activating HIF-1. The PTEN/PI3K/Akt pathway was confirmed to be a regulator of the LY6E-mediated upregulation of HIF-1 at the transcription level. LY6E expression levels were significantly higher in basal-like subtype human breast cancers than in normal breast tissues, and were strongly associated with the poor prognoses of various types of cancer patients.

## RESULTS

### LY6E upregulates HIF-1 activity

We recently developed a genetic screening method to explore novel activators of HIF-1 [24, 25]. Briefly, genetically modified NIH3T3 cells, which exhibited antibiotic resistance in a HIF-1-dependent manner, were infected with lentiviruses encoding a mouse embryo cDNA library, and then cultured in antibiotic-containing medium under normoxic conditions. cDNAs were rescued by PCR from antibiotic-resistant colonies, with the expectation that these cDNAs would have induced antibiotic resistance *via* the activation of HIF-1. Using this approach, we identified mouse lymphocyte antigen 6 complex, locus E (Ly6e) as a novel candidate activator of HIF-1. We chose to focus on the human homolog of LY6E as human and mouse LY6E displayed high degrees of amino acid sequence homologies with each other.

**Figure 1:**
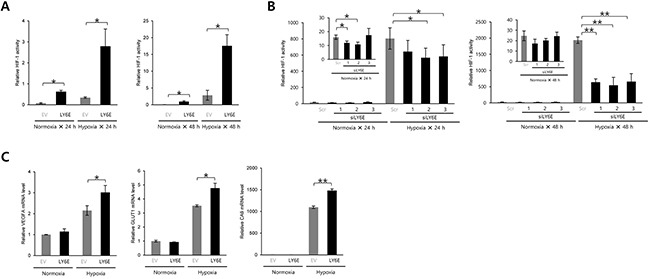
LY6E upregulated HIF-1 activity **A.** HeLa/5HRE-Luc cells were transfected with pcDNA4/LY6E (LY6E) or pcDNA4/myc-His A (empty vector: EV) and pRL-SV40, cultured under normoxic or hypoxic conditions for the indicated period, and subjected to the luciferase assay. **B.** MCF7 cells transiently transfected with p5HRE-Luc were treated with scramble-siRNA (Scr) or LY6E-siRNA (siLYE-1, 2, or 3), cultured under normoxic or hypoxic conditions for the indicated period, and subjected to the luciferase assay. **C.** HeLa cells were transiently transfected with either pcDNA4/LY6E (LY6E) or pcDNA4/myc-His A (EV), cultured under normoxic or hypoxic conditions for 24 h, and then subjected to qRT-PCR to quantify the mRNA levels of the indicated genes. Mean ± s.d. n = 3. *P < 0.05, **P < 0.001 (Student's *t*-test between the 2 indicated samples).

We performed a luciferase assay using the *5HREp-luc* reporter gene, in which five repeats of HRE functioned in the expression of *firefly* luciferase in a HIF-1-dependent manner, to determine whether LY6E induced HIF-1 activity. The forced expression of LY6E in HeLa cells, which originally displayed no detectable LY6E expression in Western blotting, resulted in the upregulation of *5HREp-luc* reporter activity under both normoxic and hypoxic conditions (Figure 1A and Supplementary Figure S1A). The MDA-MB-231 breast cancer cell line also exhibited the LY6E-induced upregulation of HIF-1 activity (Supplementary Figure S2). In contrast, the knockdown of endogenous LY6E in MCF-7 cells, a cell line that expresses high levels of endogenous LY6E, resulted in a significant decrease in HIF-1 activity (Figure 1B and Supplementary Figure S1B). Quantitative RT-PCR analyses demonstrated that the overexpression of LY6E led to an increase in the mRNA levels of various HIF-1-target genes including *VEGF*, *GLUT1*, and *CA9* in HeLa cells under hypoxic conditions (Figure 1C). Taken together, these results showed that LY6E functioned to induce HIF-1 activity.

### LY6E upregulates HIF-1α gene expression principally at the transcriptional level

**Figure 2:**
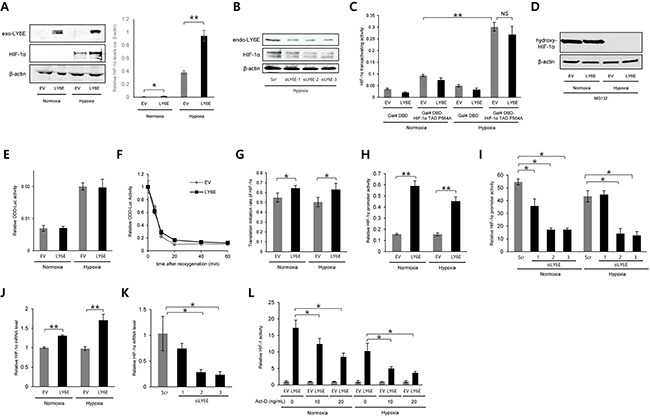
LY6E upregulated HIF-1α gene expression principally at the transcriptional level **A** and **B.** HeLa (A) or MCF7 (B) cells were transiently transfected with either pcDNA4/LY6E (LY6E) or pcDNA4/myc-His A (EV) (A) or with either scramble-siRNA (Scr) or LY6E-siRNA (siLY6E-1, 2, or 3) (B), cultured under the indicated oxygen conditions, and subjected to western blotting for the indicated proteins. The intensity of the bands was quantified using ImageJ and relative HIF-1α expression levels (vs. β-actin) are shown in the right panel (A). **C.** HeLa cells were transiently co-transfected with pG5H1bLuc, pRL-SV40, either pcDNA6/Gal4 or pcDNA6/Gal4DBD-HIF-1α TAD P564A, and either pcDNA4/LY6E or its empty vector, cultured under normoxic or hypoxic conditions for 24 h, and subjected to the luciferase assay. **D** and **E.** HeLa (D) or HeLa/ODD-Luc cells (E) were transiently transfected with either pcDNA4/LY6E or pcDNA4/myc-His A (EV) and with (E) or without (D) pRL-SV40, cultured under the indicated oxygen conditions in the presence (D) or absence (E) of 30 μM of the proteasome inhibitor, MG132, and harvested for Western blotting for the indicated proteins (D) or luciferase assay (E). **F.** HeLa/ODD-Luc cells were transfected with pRL-SV40 and either pcDNA4/LY6E (LY6E) or pcDNA4/myc-His A (EV), cultured under hypoxic conditions for 48 h, and treated with cyclohexamide (10 μg/mL) under normoxic conditions. After the reoxygenation treatment for the indicated period, cells were subjected to the luciferase assay. **G–K.** HeLa (G, H, and J) or MCF7 (I and K) cells were transiently transfected with pRL-SV40, either pcDNA4/LY6E or pcDNA4/myc-His A (EV), and either pGL3/HIF-1α 5′UTR-luc (G) or pHIF-1α promoter-luc (H and I), treated with (I and K) or without (G, H, and J) scramble-siRNA (Scr) or LY6E-siRNA (siLY6E-1, 2, or 3), cultured under either normoxic or hypoxic conditions (G-J) or under hypoxic conditions (K), and subjected to the luciferase assay (G-I) or qRT-PCR to quantify the mRNA levels of the indicated genes (J and K). **L.** HeLa/5HRE-Luc cells were transiently co-transfected with pRL-SV40 and either pcDNA4/LY6E (LY6E) or pcDNA4/myc-His A (EV), treated with indicated concentrations of Act-D, cultured under normoxic or hypoxic conditions, and subjected to the luciferase assay. Relative firefly luciferase activity to renilla luciferase activity in LY6E-expressing cells was further normalized by that in EV-transfected cells at each Act-D concentration. Mean ± s.d. n=4. *P < 0.05, **P < 0.001 (Student's *t*-test between the 2 indicated samples).

Although HIF-1 activity is regulated at multiple steps, such as the transcriptional initiation, translational initiation, and transactivation of HIF-1α, post-translational modifications to HIF-1α by PHDs and FIH-1 are known to be the most influential in regulating the stability and transactivating activity of HIF-1α, respectively. Therefore, in order to identify a critical step for the LY6E-mediated activation of HIF-1, we first investigated whether LY6E increased the expression levels and transactivating activity of the HIF-1α protein. Western blotting using HeLa cells demonstrated that the overexpression of LY6E led to a significant increase in HIF-1α protein levels under hypoxic conditions; however, whether this also occurred under normoxic conditions was unclear because the basal expression levels of HIF-1α were below the detection limits (Figure 2A). A human breast cancer cell line, MDA-MB-231, exhibited the same result that the forced expression of LY6E upregulated HIF-1α expression levels (Supplementary Figure S3). The hypoxia-mediated induction of HIF-1α expression was strongly inhibited by silencing the endogenous LY6E in MCF-7 cells, which further strengthened the importance of an increase in HIF-1α protein levels for the LY6E-mediated activation of HIF-1 (Figure 2B and Supplementary Figure S1B). On the other hand, the luciferase assay, which was performed to quantify the transactivating activity of HIF-1α using a fusion gene of the Gal4-DNA binding domain (DBD) and the transactivation domain of HIF-1α with a P564A mutation (HIF-1α TAD P564A), revealed that the transactivating activity of the HIF-1α protein, which was enhanced under hypoxic conditions, was not influenced by the forced expression of LY6E (Figure 2C).

We then examined how LY6E increased HIF-1α protein levels. Since HIF-1α expression levels are known to be principally regulated through the hydroxylation of P564 in the ODD domain by PHDs, ubiquitination by the VHL-containing E3 ubiquitin ligase, and proteolysis, we analyzed the influence of the overexpression of LY6E on these processes. Western blotting using an anti-hydroxy-HIF-1α (P564) antibody showed that LY6E did not affect the activity of the PHDs (Figure 2D). We performed a luciferase assay using the *ODD-luc* reporter gene, which expressed a fusion protein of the ODD domain (HIF-1α 548-604) with luciferase and, therefore, was useful for monitoring HIF-1α stability as luciferase bioluminescence. The overexpression of LY6E influenced neither HIF-1α stability regardless of the oxygen conditions present (Figure 2E) nor the HIF-1α half-life after a reoxygenation treatment *in vitro* (Figure 2F).

We then examined the involvement of LY6E in regulating the rate of HIF-1α synthesis at transcriptional and translational levels. To determine whether LY6E upregulated the rate of HIF-1α translation, we employed the *HIF-1α 5′UTR-luc* reporter gene, which included the internal ribosome entry site (IRES) derived from the 5′UTR of the HIF-1α gene upstream of the luciferase coding sequence and, therefore, can be used to monitor the rate of the translational initiation of HIF-1α as luciferase bioluminescence. The overexpression of LY6E significantly increased the rate of translational initiation in HeLa cells (Figure 2G); however, this increase appeared to be too small to fully explain the marked increase in HIF-1 activity by LY6E, as observed in Figure 1A. Therefore, we tested the influence of LY6E at the transcriptional level by utilizing the *pHIF-1α promoter-luc* reporter plasmid, in which the 5′-flanking sequence (5′-FS) from the *HIF-1α* gene was inserted upstream of the firefly luciferase coding sequences. The overexpression of LY6E led to a marked increase in *HIF-1α promoter-luc* reporter activity in HeLa (Figure 2H). The induction ratio appeared to be sufficient to be responsible for the LY6E-mediated increase in HIF-1 activity. The knockdown of *LY6E* in MCF-7 cells was confirmed to significantly reduce reporter activity under both normoxic and hypoxic conditions (Figure 2I). Quantitative RT-PCR confirmed that the overexpression of LY6E and silencing of endogenous LY6E resulted in significant increases and decreases in HIF-1α mRNA levels in HeLa and MCF-7 cells, respectively (Figure 2, J and K). To further confirm the involvement of the transcriptional regulation of the HIF-1α gene in the LY6E-mediated activation of HIF-1, we performed a *5HREp-luc* reporter assay with the transcriptional inhibitor, Actinomycin-D (Act-D). To exclude the direct influence of the Act-D treatment on the transcription of luciferase gene from the reporter and to only evaluate the net influence of the Act-D-dependent suppression of HIF-1α transcription on the LY6E-mediated activation of HIF-1, relative *5HREp-luc* activity, which was calculated through normalization by renilla luciferase activity, in LY6E-transfected cells was divided by that in empty vector (EV)-transfected cells at each Act-D concentration (Figure 2L). The luciferase assay revealed that Act-D reduced the LY6E-mediated activation of HIF-1, highlighting the importance of transcriptional regulation (Figure 2L). Taken together, these results indicated that LY6E mainly upregulated the transcription of the HIF-1α gene, leading to an increase in HIF-1α protein levels and HIF-1 activity.

### PTEN and PI3K/Akt signaling pathway are involved in LY6E-mediated regulation of HIF-1α transcription

**Figure 3:**
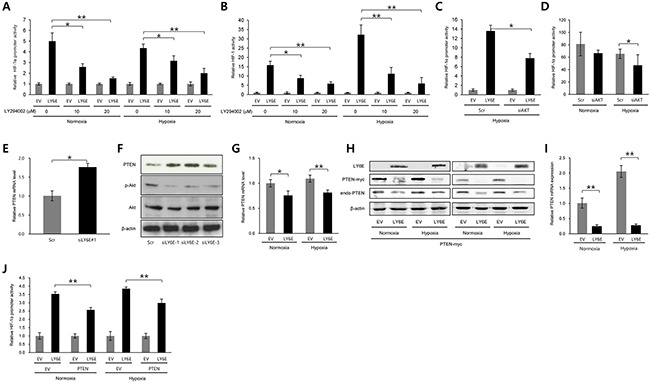
PTEN and PI3K/Akt signaling pathways were involved in the LY6E-mediated increase in HIF-1α transcription **A-F.** HeLa (A and C), HeLa/5HRE-Luc (B), or MCF7 (D-F) cells were transfected with either pcDNA4/LY6E (LY6E) or pcDNA4/myc-His A (EV) (A-C) either scramble-siRNA (Scr) or Akt-siRNA (C and D), or either scramble-siRNA (Scr) or LY6E-siRNA (siLY6E-1, 2, or 3) (E and F), and with (A, C, and D) or without (B, E, and F) pHIF-1α promoter-Luc, cultured under either normoxic or hypoxic conditions or under hypoxic conditions if not stated in the presence (A and B) or absence (C-F) of the indicated concentrations of LY294002, and subjected to the luciferase assay (A-D), qRT-PCR for PTEN mRNA levels (E), and Western blotting for the indicated proteins (F). **G**-**J.** HeLa cells were transfected with either pcDNA4/LY6E (LY6E) or pcDNA4/myc-His A (EV) and with (H-J) or without (G) either pcDNA4/PTEN-myc (PTEN) or pcDNA4/myc-His A (EV), cultured under normoxic or hypoxic conditions for 24 (G, H left, I, and J) or 48 (H right) hours, and subjected to qRT-PCR for PTEN mRNA levels (G and I), Western blotting for the indicated proteins (H), or the luciferase assay (J). Mean ± s.d. *P < 0.05, **P < 0.001 (Student's *t*-test between the 2 indicated samples).

We further investigated the molecular mechanisms underlying the LY6E-dependent upregulation of the transcriptional initiation of the HIF-1α gene. The phosphatidylinositol 3-kinase (PI3K)/Akt signaling pathway was previously shown to play an important role in both the transcriptional [20] and translational initiation [21] of the HIF-1α gene; therefore, we examined its importance in the LY6E-HIF-1 axis using the PI3K inhibitor, LY294002. The luciferase assay using either *HIF-1α promoter-luc* or *5HREp-luc* revealed that LY6E-dependent increases in luciferase bioluminescence in HeLa cells were significantly reduced by the inhibitor in a dose-dependent manner (Figure 3A and 3B). The knockdown of *Akt1* also abrogated the LY6E-mediated increase in bioluminescence from the *HIF-1α promoter-luc* reporter gene (Figure 3C). Knockdown of endogenous expression of Akt1 consistently resulted in the significant suppression of HIF-1α promoter activity in MCF7 cells (Figure 3D). Taken together, these results indicated that the PI3K/Akt signaling pathway mediated the LY6E-dependent upregulation of HIF-1α transcription and resulting activation of HIF-1.

Since it has been well established that PTEN is the most influential upstream suppressor of PI3K/Akt signaling [26], we next investigated whether PTEN participated in the LY6E-mediated regulation of HIF-1 activity. The knockdown of endogenous LY6E led to a significant increase in PTEN mRNA levels in MCF-7 cells (Figure 3E). Western blotting using anti-PTEN, anti-phosphorylated-Akt, and anti-Akt revealed that the knockdown of LY6E in MCF-7 cells increased PTEN levels and decreased the phosphorylation of the Akt protein (Figure 3F). On the other hand, the overexpression of LY6E reduced the mRNA and protein levels of the PTEN gene under both normoxic and hypoxic conditions (Figure 3, G and H). Exogenously as well as endogenously expressed PTEN (PTEN-myc) was down-regulated by the forced expression of LY6E at both the mRNA and protein levels, suggesting that LY6E reduced PTEN mRNA levels by recognizing the coding region of PTEN (Figure 3, H and I). The *HIF-1α promoter-luc* reporter assay was performed following the transient transfection of either EV or LY6E and either EV or PTEN expression vectors in order to directly determine the participation of PTEN expression in the mechanism by which LY6E upregulated HIF-1α transcription (Figure 3J). Introduction of the PTEN expression vector significantly abrogated the *HIF-1α promoter-luc* reporter activity induced by the overexpression of LY6E in HeLa cells. Taken together, these results indicated that LY6E positively regulated HIF-1α expression at the transcriptional initiation level by reducing the levels of PTEN mRNA and consequently activating the PI3K/Akt pathway.

### LY6E activates HIF-1, induces angiogenesis, and consequently accelerates tumor growth

**Figure 4:**
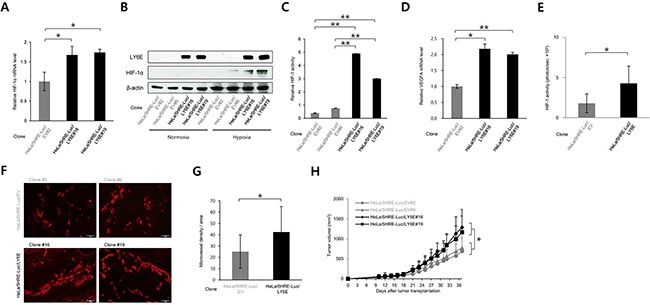
LY6E activated HIF-1, induced angiogenesis, and consequently accelerated tumor growth **A**-**D.** Stable transfectants of HeLa/5HRE-Luc/EV and HeLa/5HRE-Luc/LY6E cells were cultured under either normoxic or hypoxic conditions or under hypoxic conditions if not stated, and subjected to qRT-PCR for the indicated genes (A and D), Western blotting for the indicated proteins (B), and the luciferase assay. (n = 3). **E.** Bioluminescent intensity from tumor xenografts with the indicated cells was acquired with the IVIS-SPECTRUM imaging system and normalized by each tumor volume 23 days after tumor transplantation into 6 nude mice per one cell line (n = 6). **F** and **G.** Tumor xenografts of the indicated cells were surgically excised 37 days after cancer cell transplantation and subjected to an immunohistochemical analysis using an anti-CD31 antibody (F). Microvessel density detected as CD31-positive cells per one field in F was quantified (3 fields in each of 10 representative xenografts, n = 30). **H.** Growth curves of tumor xenografts after the transplantation of HeLa/5HRE-Luc/EV #2, 6 and HeLa/5HRE-Luc/LY6E#16, 19 cells into 10 nude mice per one cell line. Mean ± s.d. *P < 0.05, **P < 0.01, (Student's *t*-test between the 2 indicated samples).

We next investigated whether the LY6E-HIF-1 axis promoted tumor growth *in vivo*. We stably transfected HeLa/5HRE-Luc cells, which expressed luciferase under the control of HIF-1, with either the LY6E expression vector (HeLa/5HRE-Luc/LY6E clone #16, 19) or its empty vector (HeLa/5HRE-Luc/EV clone #2, 6). The forced expression of LY6E was confirmed to induce the expression of HIF-1α mRNA and the HIF-1α protein, upregulate HIF-1 activity, and consequently induce the expression of HIF-1-target genes in the stable transfectants (Figure 4A-4D and Supplementary Figure S4A). Optical imaging experiments confirmed that the forced expression of LY6E resulted in high levels of HIF-1 activity during tumor growth both *in vivo* and *in vitro* (Figure 4E and Supplementary Figure S4B).

We then examined the influence of LY6E on tumor vasculature because we confirmed that LY6E significantly induced the expression of pro-angiogenic factors, such as VEGF and PDGFB (Figure 1C and 4D and Supplementary Figure S4A). An immunohistochemical analysis against a marker of endothelial cells, CD31, demonstrated that microvessel density was significantly greater in HeLa/5HRE-Luc/LY6E tumors than in HeLa/5HRE-Luc/EV tumors (Figure 4, F and G and Supplementary Figure S5). The tumor growth assay revealed that the forced expression of LY6E significantly enhanced the growth of HeLa/5HRE-Luc/LY6E tumors more than HeLa/5HRE-Luc/EV tumors xenografted in immunodeficient mice (Figure 4H). These *in vivo* results collectively indicated that the aberrant expression of LY6E positively regulated HIF-1 activity, induced angiogenesis, and eventually accelerated tumor growth.

### Clinical implications of LY6E in human cancer

**Figure 5:**
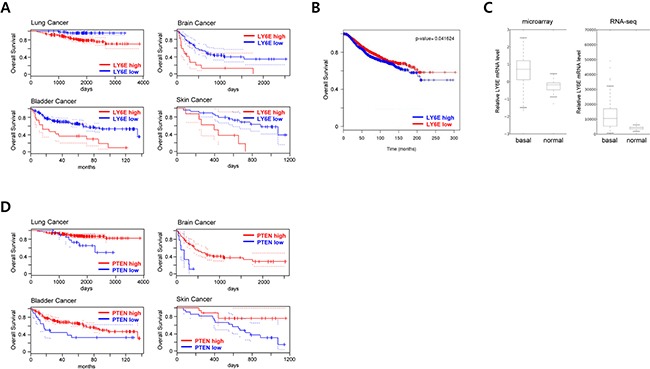
Clinical implication of LY6E in human cancer **A.** PrognoScan database-based Kaplan-Meier analyses of the overall survival of 204 lung (data set: GSE31210; stage I-II lung adenocarcinomas) [46], 165 bladder (data set: GSE13507; histologically diagnosed transitional-cell carcinoma) [47], 74 brain (data set: GSE4412-GPL96; grade III and IV gliomas) [48], and 38 skin (data set: GSE19234; stage III and IV metastatic melanomas) [49] cancer patients stratified by high (red) and low (blue) LY6E expression levels (lung, high: n = 141, low: n = 63; bladder, high: n = 19, low: n = 146; brain, high: n = 17, low: n = 57; and skin, high: n = 8, low: n = 30). **B.** A BreastMark database-based Kaplan-Meier analysis of the overall survival of 2091 breast cancer patients stratified by high (blue) and low (red) LY6E expression levels. **C.** LY6E expression levels in basal-like breast cancer tissues (n=98) and normal breast tissues (n=22) from TCGA 2012 data. **D.** PrognoScan database-based Kaplan-Meier analyses of the overall survival of the same data set as A stratified by high (red) and low (blue) PTEN expression levels.

To confirm our results in human cancers, we analyzed the relationship between LY6E expression levels and poor prognoses using the PrognoScan database. We confirmed that the expression of LY6E positively correlated with poor overall survival rates in several human cancer patients including lung, bladder, brain, and skin cancers (Figure 5A and Supplementary Figure S6). The same correlation was confirmed in breast cancer patients in another bioinformatics analysis using the BreastMark database (Figure 5B).

High expression levels of LY6E have been reported in basal-like breast cancer cell lines and have been associated with pulmonary breast cancer metastasis [12, 14]. These findings prompted us to further analyze the expression levels of LY6E in basal-like breast cancer tissues. The microarray and RNA-seq data sets from 2012 TCGA [27] showed significantly higher expression levels of LY6E in basal-like breast cancer than in surrounding normal breast tissues (Figure 5C).

Based on the result that LY6E expression levels were inversely correlated with those of PTEN (Figure 3E-3I) and with the poor overall survival of cancer patients (Figure 5, A and B), we hypothesized that expression levels of PTEN in malignant tumors should be correlated with the poor prognosis of cancer patients. PrognoScan-based database analyses on PTEN using the same data set as in Figure 5A (GSE31210, GSE13507, GSE4412-GPL-96, and GSE19234 for patients with lung, bladder, brain, and skin cancers, respectively) actually revealed that patients with low intratumoral PTEN expressions exhibited poor overall survival (Figure 5D and Supplementary Figure S7).

## DISCUSSION

In the present study, we identified LY6E as an inducer of HIF-1α expression and defined the novel mechanistic and functional linkages of the LY6E-HIF-1 axis with angiogenesis and growth of malignant solid tumors.

Our *in vitro* studies revealed that the aberrant overexpression of LY6E upregulated the transcription of the HIF-1α gene by reducing PTEN expression at the mRNA level and subsequently activating the PI3K/Akt signaling pathway. However, some missing links exist in the mechanism. It remains unclear how LY6E reduced PTEN mRNA levels. We herein obtained results to show that LY6E overexpression decreased the levels of not only endogenously, but also exogenously expressed PTEN mRNA. PTEN mRNA levels were consistently reported to be regulated by some genes, such as IGF2BP1 [28] and TGF-β1 through their direct actions on the PTEN coding region [29]. Future studies to investigate the possibility that LY6E or its downstream factor is involved in the downregulation of PTEN at the post-transcriptional level are warranted. The mechanisms of actions of the activated PI3K/Akt signaling pathway to the HIF-1α gene are also still unclear. The PI3K/Akt signaling pathway has been shown to positively affect the translational as well as transcriptional initiation of the HIF-1α gene. Our luciferase assay using the *HIF-1α 5′UTR-luc* reporter gene consistently showed that the translational initiation of HIF-1α was significantly upregulated following the overexpression of LY6E. Identifying the critical step is crucial for fully understanding the molecular mechanisms underlying the LY6E-mediated activation of HIF-1, which will, in turn, assist in developing inhibitors of the LY6E-HIF-1 axis for cancer therapy.

Tumor angiogenesis has been closely associated with tumor growth, hematogenous metastasis, and the poor prognoses of cancer patients [30]. We demonstrated that the induction of HIF-1 activity in tumor xenografts as a result of the aberrant expression of LY6E enhanced angiogenesis and malignant tumor growth. Moreover, we confirmed that LY6E expression levels in malignant tumors correlated with the poor overall survival of patients with various cancers including lung, bladder, brain, and skin cancers. The expression levels of LY6E were significantly higher in basal-like breast cancer than in surrounding normal breast tissues in the TCGA data analysis. Taken together, these results justified exploiting LY6E as a prognostic marker as well as a therapeutic target for cancers.

Basal expression levels of LY6E have been reported to be high in normal T cells. Therefore, we would like to suggest the possibility that the impact of the aberrant overexpression of LY6E on the HIF-1-mediated malignant progression demonstrated in the present study can be observed only in types of malignant cells other than malignant T cells. More importantly, we suggest that it might be better to pay attention to the side effects affecting normal T cells when we exploit LY6E as a target for cancer therapy.

It has not yet been elucidated how LY6E expression levels are upregulated in some types of cancers. Previous studies provided some suggestions. Five out of 47 primary breast tumors and 10 out of 18 breast cancer cell lines were reported to have a high level copy number of the chromosome 8q24 region. This amplicon, which extends from 139.3 to 144.8 Mb (8q24.3), encodes 8 genes from LY6 family members including LY6E [31]. Furthermore, type I interferons, such as IFN-α, have been reported to strongly enhance LY6E mRNA levels in human cancer cells because of the presence of the IFN-α-stimulated response element (ISRE) on the upstream sequence of LY6E [32–34]. On the other hand, GATA3, the low expression of which has been linked to the malignant progression of breast cancers, has been shown to negatively regulate LY6E expression in breast cancer cells [14]. Based on accumulating evidence, elucidating the mechanisms underlying the aberrant overexpression of LY6E in cancer cells will provide an insight into the development of rational strategies that target the LY6E-HIF-1 axis for cancer therapy.

## MATERIALS AND METHODS

### Cell culture and reagents

NIH/3T3, HeLa, MCF7, and MDA-MB-231 cell lines were purchased from the American Type Culture Collection and maintained in 10% FBS-Dulbecco's modified Eagle's medium. Cells were incubated in a well humidified incubator with 5% CO_2_ and 95% air for the normoxic condition. Cells were incubated in the Bactron Anaerobic Chamber, BACTLITE-2 (Sheldon Manufacturing, Comelius, OR) and in RUSKIN INVIVO2 500 (The Baker Company, Sanford, FL) for the hypoxic condition at <0.02% O_2_ and 1% O_2_, respectively. Stock solutions of LY294002 (Cell signaling), Actinomycin-D (Act-D), and cycloheximide (Nacalai Tesque) were prepared in DMSO (10 or 20 μM, 10 or 20 ng/mL, and 10 mg/mL, respectively). Double strand RNAs for the transient silencing of LY6E (silencer Select Validated siRNA, Cat. No. SR302746A: 5′-cccagaaggcgucaauguuggugug-3′, SR302746B: 5′-gcuuuggugccucaaauaaauacag-3′, and SR302746C: 5′-gcuccgaccaggacaacuacugcgu-3′) and the universal scrambled negative control (Cat. No. SR30004: sequence information is not disclosed) were purchased from Origene (Origene Technologies, Inc., Rockville, MD).

### Plasmid constructs

To construct pcDNA4/LY6E, the cDNA encoding human *LY6E* gene was amplified from the cDNA of HeLa cells and inserted between the BamHI and XhoI sites of pcDNA4/myc-His A (Invitrogen, Carlsbad, CA). To construct pEF/SV40p-ODD-Luc encoding the ODD-Luc reporter gene, the DNA fragment encoding the *SV40p-ODD-luc* reporter gene was prepared by digesting pGL3/ODD-Luc with KpnI and XbaI, and inserted between the corresponding sites of pEF/myc/cyto (Invitrogen). The plasmid p5HRE-Luc was constructed as described previously [35]. To construct pHIF-1α promoter-luc and pGL3/HIF-1α 5′UTR-luc, the DNA fragments of the HIF-1α promoter (−572 - +32) and HIF-1α 5′UTR (+1 - +284) were amplified from the genomic DNA of HeLa cells and inserted between the BglII and HindIII sites and also between the HindIII and NcoI sites of the pGL3 promoter vector (Promega), respectively. To construct pcDNA4/PTEN-myc, the cDNA encoding the human PTEN gene was amplified from the cDNA of MCF7 cells and inserted between the XhoI and XbaI sites of pcDNA4/myc-His A (Invitrogen). To construct pcDNA6/Gal4DBD-HIF-1α TAD P564A, which expresses a fusion protein of Gal4 DBD and HIF-1α TAD with a P564A mutation, a DNA fragment encoding Gal4 DBD prepared from the GalA expression vector and that encoding HIF-1α CTAD P564A (HIF-1α 531a.a - 826a.a. with P564A mutation) were inserted into the HindIII and EcoRI sites and between the EcoRI and XhoI sites of pcDNA6/V5-His A (Invitrogen), respectively. The plasmid pG5H1bLuc, containing five Gal4-binding sites upstream of the adenovirus E1b promoter and firefly luciferase CDS, was constructed as described previously [22, 36].

### Stable transfectants

HeLa and MDA-MB-231 cells were stably transfected with pEF/SV40p-ODD-Luc (for HeLa/ODD-Luc) [37], p5HRE-Luc (for HeLa/5HRE-Luc and MDA-MB-231/5HRE-Luc) [35, 38], or a combination of p5HRE-Luc and either pcDNA4/myc-His A (Invitrogen; for HeLa/5HRE-Luc/EV [clone #2, 6]) or pcDNA4/LY6E (for HeLa/5HRE-Luc/LY6E [clone #16, 19]). Cells were then cultured for 10-14 days in culture medium containing the corresponding antibiotic to select antibiotic-resistant stable transfectants. The resultant colonies were isolated and established as clones. Representative clones showing expected and reasonable activities were used in the present study.

### Screening of LY6E as an upstream activator of HIF-1

LY6E was isolated from the mouse embryo cDNA library (Clontech) as a potential activator of HIF-1 through genetic screening experiments [24, 25].

### Luciferase assay and western blotting

Twenty-four hours after cells (2 × 10^4^ cells/well in a 24-well plate for the luciferase assay and 2 × 10^5^ cells/well in a 6-well plate for Western blotting) were transfected with the indicated plasmids, they were treated under normoxic (20% O_2_) and hypoxic (<0.02 or 1% O_2_) conditions for the luciferase assay and Western blotting, respectively, for the indicated periods, and harvested in 100 μL Passive Lysis Buffer (Promega) for the luciferase assay or 150 μL Cell Lytic Buffer (Sigma-Aldrich) for western blotting. The luciferase assay was performed using the dual luciferase assay kit according to the manufacturer's instructions (Promega). The plasmid, pRL-SV40 (Promega), was used as an internal control to calculate relative luciferase activity. Western blotting was performed using anti-HIF-1α (BD Bioscience for human HIF-1α, Cat# 610959), anti-LY6E (Abnova Cat# H00004061-D01P), anti-PTEN (Cell Signaling Cat# 9559), anti-hydroxy-HIF-1α (Pro564; Cell Signaling), anti-β-actin (BioVision Cat# 3598-100), anti-Akt (Cell Signaling Cat# 9272), and anti-phospho-Akt (Ser473) (Cell Signaling Cat# 9271) antibodies as the primary antibodies, anti-mouse and anti-rabbit IgG horseradish peroxidase-linked whole antibodies (GE Healthcare) as the secondary antibodies, and the ECL Plus Western Blotting Detection system (GE Healthcare) for detection according to the manufacturer's instructions.

### qRT-PCR

After cells (2 × 10^5^ cells/well in a 6-well-plate) were treated as described in the figure legends, total RNA was extracted and subjected to reverse transcription as described previously [24, 25]. The mRNA level of the indicated gene was quantified by the qRT-PCR technique using the Thermal Cycler Dice Real Time System (TP-800; Takara Bio) with the SYBR Premix Ex Taq kit (Takara Bio) and commercial primers (TaKaRa Primer Set ID HA074624 for HIF-1α mRNA, HA160790 for VEGFA mRNA, HA154269 for PDGF-β mRNA, HA126121 for GLUT1 mRNA, HA176356 for CA9 mRNA, HA112155 for PTEN mRNA, HA139090 for LY6E mRNA, HA067803 for human β-actin mRNA) according to the manufacturer's instructions. β-actin mRNA levels were used as a normalizer.

### *In vivo* experiments

Cancer cell suspensions (100 μL of 3 × 10^5^ cells in PBS) were subcutaneously transplanted into the right hind legs of athymic nude mice (BALB/c nu/nu: SLC, Japan). Tumor volumes were calculated as 0.5 × length × width^2^, and compared with the initial value to calculate relative tumor volume. Representative images of tumor-bearing mice were taken with the IVIS spectrum *in vivo* imaging system (Caliper) as described previously [35, 38–42].

### Immunohistochemical analysis

Frozen sections of tumor xenografts were treated with the purified rat anti-mouse CD31 antibody (BD Pharmingen Cat# 550274, × 100 dilution) and Alexa Fluor 594 goat anti-rat IgG (Invitrogen, x 2,000 dilution) as described previously [43].

### Analysis of prognoscan, breastmark, and TCGA data

The relationship between LY6E expression and overall survival in cancer patients was evaluated by an analysis of the PrognoScan [44] and BreastMark [45] databases. Briefly, patients were divided into two groups according to LY6E expression levels in their tumors at all possible cut-off points. The risk differences of any two groups were then calculated by the log-rank test. The cut-off point giving the most significant *P* value was selected and demonstrated in the present study. The TCGA dataset is described in [27] and was downloaded from the TCGA data portal site (https://gdc.cancer.gov) for the next analysis.

### Ethics of research using animals

All animal experiments were approved by the Animal Research Committee of Kyoto University, and performed according to guidelines governing animal care in Japan.

### Statistical analyses

The significance of differences between 2 independent subjects was determined using the Student's *t*-test. A *P* value < 0.05 was considered to be significant.

## SUPPLEMENTARY MATERIALS FIGURES


