# Supplementary material for: LY6E: a conductor of malignant tumor growth through modulation of the PTEN/PI3K/Akt/HIF-1 axis
Source: Oncotarget. 2016 Aug 29;7(40):65837–48. doi: 10.18632/oncotarget.11670 (PMC5323196; doi:10.18632/oncotarget.11670)
Supplement: Supplementary file 1 [file oncotarget-07-65837-s001.pdf]

LY6E: a conductor of malignant tumor growth through modulation of the PTEN/PI3K/Akt/HIF-1 axis

SUPPLEMENTARY FIGURES

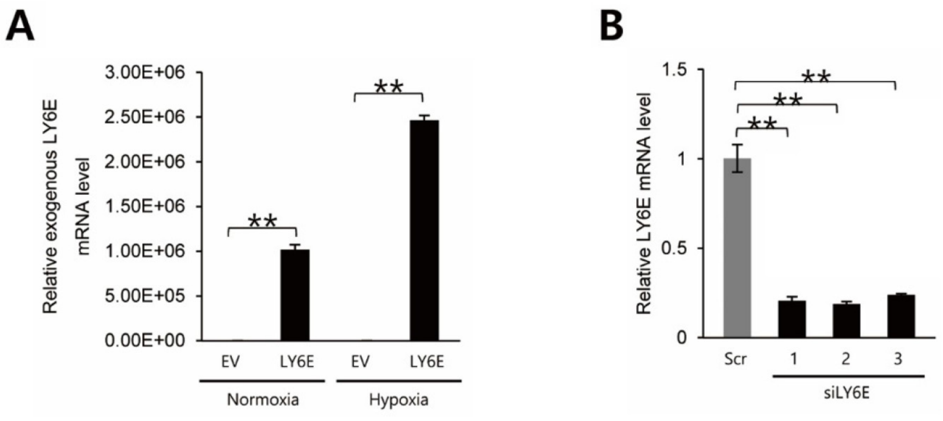

Supplementary Figure S1: Overexpression and knockdown of LY6E. A. HeLa cells were transiently transfected with pcDNA4/LY6E (LY6E) or pcDNA4/myc-His A (EV), cultured under normoxic or hypoxic conditions for 24 h, and subjected to qRT-PCR using primers for exogenous *LY6E*. **B.** MCF7 cells were treated with scramble-siRNA (Scr) or LY6E-siRNA (siLY6E-1, 2, 3), cultured under hypoxic conditions for 24 h, and subjected to qRT-PCR to quantify the mRNA levels of LY6E and HIF-1α genes. Mean ± s.d. n = 4, $^{**}P < 0.01$ (Student's *t*-test).

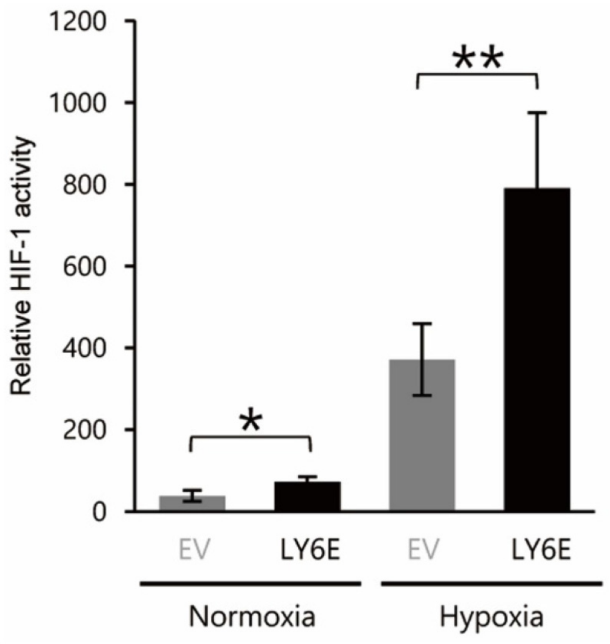

Supplementary Figure S2: Activation of HIF-1 by LY6E overexpression. MDA-MB-231/5HRE-Luc cells were transfected with pcDNA4/LY6E (LY6E) or pcDNA4/myc-His A (EV), cultured under normoxic or hypoxic conditions for 48 h, and subjected to a dual luciferase assay. Mean ± s.d. n = 4, $^{*}P < 0.05$, $^{**}P < 0.01$ (Student's *t*-test).

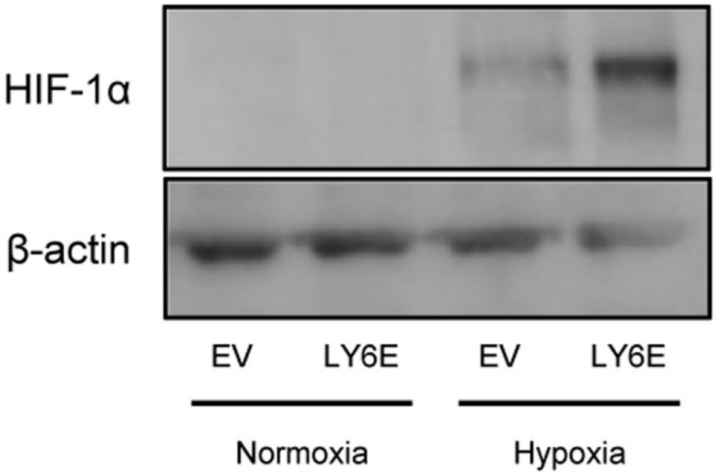

Supplementary Figure S3: LY6E-mediated upregulation of HIF-1α protein levels. MDA-MB-231 and A549 cells were transiently transfected with either pcDNA4/LY6E (LY6E) or pcDNA4/myc-His A (EV), cultured under the indicated oxygen conditions, and subjected to Western blotting for the indicated proteins.

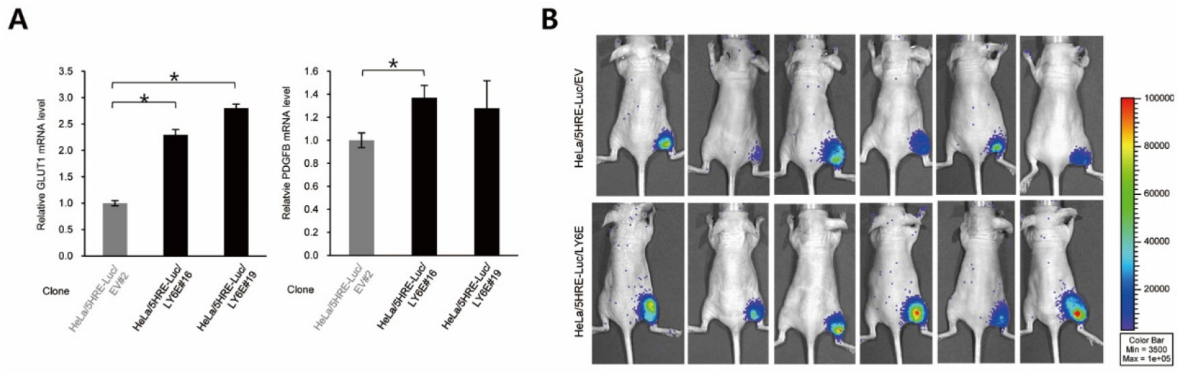

Supplementary Figure S4: LY6E-mediated activation of HIF-1 and pro-angiogenic characteristics of HeLa/5HRE-Luc/LY6E stable transfectants. A. Stable transfectants of HeLa/5HRE-Luc/EV and HeLa/5HRE-Luc/LY6E cells were cultured under hypoxic conditions and subjected to qRT-PCR for the indicated genes. Mean ± s.d. n = 4, $*P < 0.05$ (Student's *t*-test). **B.** *In vivo* bioluminescence images were acquired 23 days after the transplantation of HeLa/5HRE-Luc/EV#2 and HeLa/5HRE-Luc/LY6E#16 cells into 6 nude mice per one cell line to monitor HIF-1 activities in tumor xenografts.

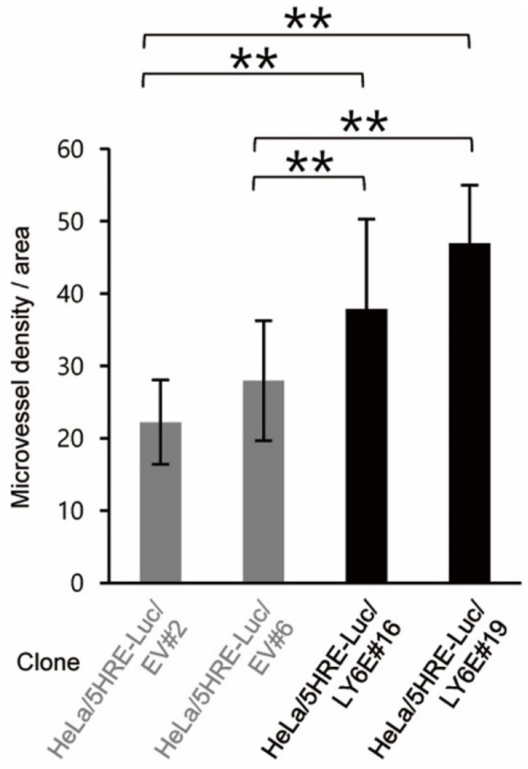

Supplementary Figure S5: Increases in microvessel density by the forced expression of LY6E. Tumor xenografts of the indicated cells were surgically excised 37 days after cancer cell transplantation and subjected to an immunohistochemical analysis using an anti-CD31 antibody. Microvessel density detected as CD31-positive cells per one field was quantified (3 fields in each of 10 representative xenografts, n = 30). Mean ± s.d. **$P < 0.01$ (Student's t-test).

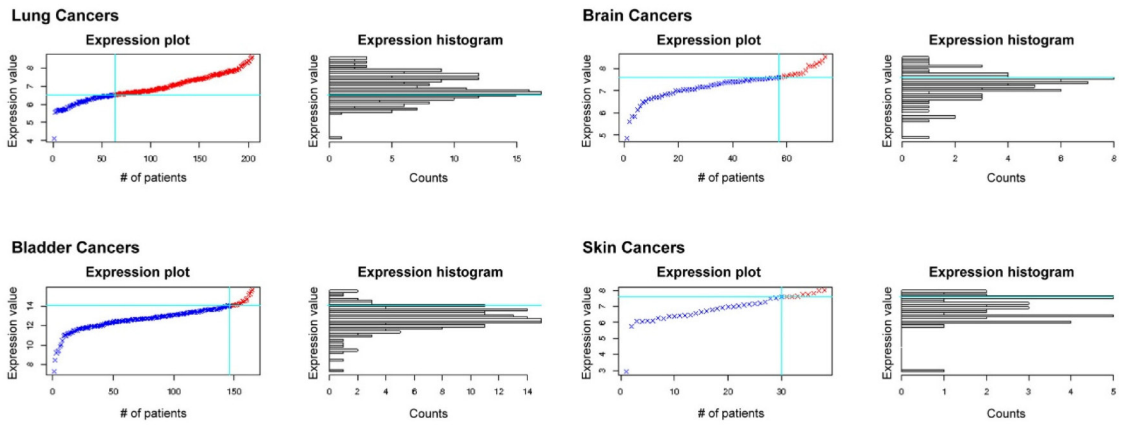

Supplementary Figure S6: Expression plots and expression histograms associated with the PrognoScan database-based analyses in Figure 5A. Cutoff values between high and low expression levels of intratumoral LY6E are represented as cyan lines in each expression plot and expression histogram of PrognoScan database-based Kaplan-Meier analysis of the overall survival of 204 lung, 165 bladder, 74 brain, and 38 skin cancer patients in Figure 5A.

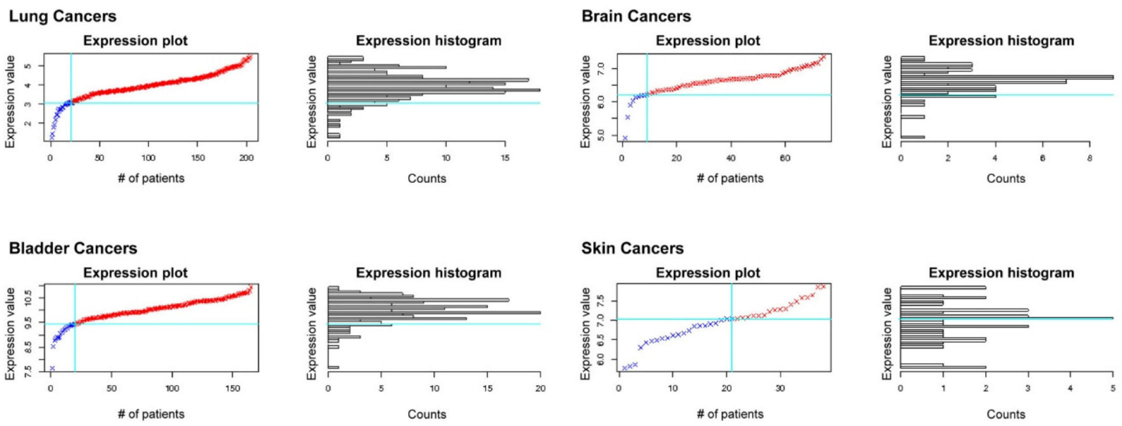

Supplementary Figure S7: Expression plots and expression histograms associated with the PrognoScan database-based analyses in Figure 5D. Cutoff values between high and low expression levels of intratumoral PTEN are represented as cyan lines in each expression plot and expression histogram of PrognoScan database-based Kaplan-Meier analysis of the overall survival of 204 lung, 165 bladder, 74 brain, and 38 skin cancer patients in Figure 5D.
